# Prevalence and Determinants of Neck Pain Among Laboratory Technicians and Clerical Staff in a Tertiary Care Hospital: A Cross-Sectional Questionnaire-Based Study

**DOI:** 10.7759/cureus.106142

**Published:** 2026-03-30

**Authors:** Palagiri Ajay Kumar Reddy, Gils Thampi, Nagakumar J S, Manoj K Ramachandraiah

**Affiliations:** 1 Orthopaedics, Sri Devaraj Urs Medical College (SDUMC), Kolar, IND

**Keywords:** clerical staff, cross-sectional study, ergonomics, laboratory technicians, musculoskeletal disorders, neck pain

## Abstract

Introduction

Neck pain is a leading cause of musculoskeletal disability worldwide, significantly diminishing productivity and quality of life. Occupational risk factors such as prolonged static postures, repetitive upper-limb movements, and poor ergonomic practices are well documented. In hospital settings, laboratory technicians and clerical staff are particularly vulnerable due to sustained task-specific activities and extended periods of sitting. Despite this, these groups remain underrepresented in occupational health research. The primary objective of this study was to determine the prevalence of neck pain among laboratory technicians and clerical staff in a tertiary care hospital, while the secondary objective was to identify associated occupational and individual risk factors.

Materials and methods

A cross-sectional, questionnaire-based study was conducted at R.L. Jalappa Hospital and Research Centre, Kolar, from July to August 2025. The study population consisted of 100 participants, evenly divided between laboratory technicians (n = 50) and clerical staff (n = 50), each with a minimum employment duration of six months. Data were collected using a structured, pretested questionnaire designed to capture demographic information, occupational exposures, ergonomic practices, and history of neck pain. Pain intensity was measured using the Visual Analog Scale (VAS), while functional disability was assessed with the validated Neck Disability Index (NDI). Statistical analyses were performed using IBM SPSS Statistics Version 26 (IBM Corp., Armonk, NY, USA). Chi-square tests and multivariate logistic regression models were employed to identify independent predictors of neck pain, with statistical significance set at p < 0.05.

Results

The overall 12-month prevalence of neck pain was 71% (71/100; 95% CI: 61.9%-79.1%). When stratified by occupational group, the prevalence was significantly higher among laboratory technicians (42/50; 84%) compared to clerical staff (29/50; 58%) (p = 0.002). The mean Neck Disability Index (NDI) score was 18.5 ± 8.2, indicating moderate functional impairment. Multivariate analysis identified female gender (adjusted odds ratio (aOR) = 2.4; 95% confidence interval (CI): 1.3-4.5; p = 0.007), engagement in static postures exceeding four hours daily (aOR = 4.1; 95% CI: 2.1-8.0; p < 0.001), body mass index (BMI) greater than 25 kg/m² (aOR = 1.9; 95% CI: 1.0-3.6; p = 0.04), and low ergonomic awareness (aOR = 2.2; 95% CI: 1.2-4.1; p = 0.01) as significant independent predictors of neck pain.

Conclusion

The findings indicate a high prevalence of neck pain among laboratory technicians and clerical staff, with significant associations to prolonged static postures and poor ergonomic practices. Implementing targeted ergonomic interventions, encouraging regular breaks, and providing workplace health education programs may help reduce neck pain and improve occupational health outcomes.

## Introduction

Neck pain is one of the most common musculoskeletal conditions worldwide and is a leading cause of years lived with disability globally [1]. Recent global burden of disease assessments emphasize its significant impact on occupational disability and productivity loss [2]. Occupational factors play a critical role in the onset and persistence of neck pain. Specifically, prolonged static postures, repetitive upper-limb movements, and poor ergonomic conditions have been strongly associated with cervical strain [3,4]. Workers engaged in visually intensive tasks requiring sustained neck flexion are particularly vulnerable [5]. Occupational groups within healthcare settings frequently encounter these risk factors. Laboratory technicians are often exposed to task-specific activities requiring sustained forward head posture with limited postural variation [6]. Similarly, clerical personnel are exposed to prolonged computer use and static sitting, often at workstations with suboptimal ergonomic design [7].

Prior research involving computer users and healthcare workers has demonstrated significant correlations between extended screen time, static postures exceeding four hours daily, and chronic neck pain [8,9]. Additionally, female sex and elevated body mass index have been identified as notable individual risk factors [10]. Psychosocial stressors and insufficient ergonomic awareness also contribute to the development of symptoms in occupational settings [11]. Despite recognition of these risk factors, data from India on the prevalence of neck pain among laboratory technicians and clerical staff in tertiary care hospitals remain scarce [12]. Establishing baseline prevalence rates and identifying modifiable risk factors are essential for developing effective ergonomic and occupational health interventions.

The primary objective of this study was to determine the prevalence of neck pain among laboratory technicians and clerical staff at a tertiary care hospital. The secondary objective was to identify associated occupational and individual risk factors. It was hypothesized that prolonged static posture, poor ergonomic practices, and selected individual factors would be significantly associated with neck pain.

## Materials and methods

This cross-sectional observational study, employing a questionnaire-based approach, was conducted at R.L. Jalappa Hospital and Research Centre, Kolar, from July to August 2025. The study protocol received approval from the Central Ethics Committee of Sri Devaraj Urs Academy of Higher Education and Research, Kolar, India (SDUAHER/R&D/CEC/SDUMC-PG/165/NF/-2025-26) and adhered to the ethical standards outlined in the Declaration of Helsinki. Participation was entirely voluntary, with all participants providing written informed consent prior to inclusion. Confidentiality of personal and occupational information was strictly maintained throughout the study.

Eligibility criteria included full-time laboratory technicians and clerical staff employed for at least six months, aged between 18 and 60 years. Exclusion criteria comprised a history of cervical spine trauma, previous cervical spine surgery, inflammatory or systemic musculoskeletal conditions, recent acute neck injury, and pregnancy. As this was a time-bound cross-sectional study, all eligible participants available during the study period were included, and no formal sample size calculation was conducted. A total of 100 participants (100%) were recruited through convenience sampling, comprising 50 laboratory technicians (50%) and 50 clerical staff members (50%). This method was chosen due to feasibility and accessibility within the study period; however, it may introduce selection bias and limit the generalizability of the findings.

Data were collected using a structured, pretested questionnaire specifically developed for this study based on relevant literature and clinical expertise. The instrument was self-designed and included predefined domains covering demographic characteristics, occupational exposure, ergonomic practices, lifestyle factors, and history of neck pain. Content validity was established through expert review, and the questionnaire was pilot-tested among 10 healthcare workers to ensure clarity and feasibility before final administration. Formal reliability testing was not conducted, which may limit measurement consistency. The instrument comprised six sections: (A) demographic characteristics, (B) occupational history, (C) ergonomic practices, (D) lifestyle and physical activity factors, (E) neck pain history, and (F) functional disability assessment. The demographic section captured variables such as age, gender, height, weight, body mass index (BMI), educational attainment, marital status, and dominant hand.

The occupational history section recorded total years of professional experience, duration in the current position, daily working hours, frequency of overtime, break schedules, types of work tasks (including microscope use, pipetting, typing, filing, and telephone use), and predominant working postures. The ergonomic practices section evaluated workstation characteristics, including chair type and adjustability, monitor height relative to eye level, keyboard and mouse placement, availability of document holders, and prior ergonomic training or awareness of proper posture.

Lifestyle factors documented included exercise frequency, smoking status, alcohol consumption, and sleep duration and quality. The neck pain history section assessed the 12-month prevalence, duration, and frequency of symptoms, as well as aggravating and alleviating factors and treatment history. Pain intensity was measured using the Visual Analog Scale (VAS), a validated instrument ranging from 0 (no pain) to 10 (worst imaginable pain) [13]. The presence of neck pain was determined based on self-reported symptoms over the past 12 months. The VAS was used exclusively to assess pain intensity among individuals reporting neck pain and was not included as a variable in the primary statistical or regression analyses.

Functional disability assessment

Functional disability related to neck pain was assessed using the Neck Disability Index (NDI), a validated instrument specifically designed to measure neck-related functional impairment [5]. Authorization to use the NDI questionnaire was obtained through the MAPI Research Trust Electronic Patient-Reported Outcome Value in Drug Evaluation (ePROVIDE) platform for academic research purposes. The NDI consists of 10 items, each scored on a scale from 0 to 5, resulting in a total score ranging from 0 to 50. Scores were interpreted as follows: no disability (0-4), mild disability (5-14), moderate disability (15-24), severe disability (25-34), and complete disability (≥35), with higher scores indicating greater functional impairment.

Statistical analysis

Data analysis was performed using IBM SPSS Statistics for Windows, Version 26.0 (IBM Corp., Armonk, NY, USA). Continuous variables are reported as means with their corresponding standard deviations, while categorical variables are presented as frequencies and percentages. Comparative analyses between laboratory technicians and clerical personnel were conducted using the chi-square test for categorical variables and the independent samples t-test for continuous variables. Logistic regression analysis was employed to identify independent predictors of neck pain, adjusting for potential confounding factors. Variables with clinical relevance, as well as those found to be statistically significant in univariate analysis, were included in the multivariate logistic regression model. Model fit was assessed using appropriate goodness-of-fit measures. Statistical significance was set at a p-value of less than 0.05.

## Results

The study included a total of 100 participants. The mean age of the sample was 32.4 ± 7.2 years, and females comprised 58/100 (58%) of the study population. The overall 12-month prevalence of neck pain was 71% (71/100; 95% CI: 61.9%-79.1%).

The demographic and occupational characteristics of the study population are summarized in Table 1. Clerical personnel had a significantly higher mean age than laboratory technicians (34.6 ± 7.5 years vs. 30.2 ± 6.5 years; p = 0.01). The gender distribution was similar between the two groups, with females comprising 32/50 (64%) of clerical staff and 26/50 (52%) of laboratory technicians (p = 0.22). Likewise, the prevalence of a body mass index (BMI) over 25 kg/m² did not differ significantly between clerical staff and laboratory technicians (18/50; 36% vs. 22/50; 44%; p = 0.42). In contrast, a significantly larger proportion of clerical employees reported more than five years of occupational experience compared to laboratory technicians (28/50; 56% vs. 12/50; 24%; p < 0.01).

**Table 1:** Demographic and Occupational Characteristics of Study Participants (N = 100) Data are presented as mean ± standard deviation or number (percentage). BMI: body mass index; χ²: chi-square test statistic; t: independent t-test statistic. A p-value < 0.05 was considered statistically significant.

Characteristic	Laboratory Technicians (n = 50)	Clerical Staff (n = 50)	Test Statistic	p-value
Age (years)	30.2 ± 6.5	34.6 ± 7.5	t = 3.21	0.01
Female, n (%)	32 (64%)	26 (52%)	χ² = 1.48	0.22
BMI >25 kg/m², n (%)	18 (36%)	22 (44%)	χ² = 0.65	0.42
Experience >5 years, n (%)	12 (24%)	28 (56%)	χ² = 10.24	<0.01

The prevalence and functional disability associated with neck pain are presented in Table 2. The 12-month prevalence of neck pain was significantly higher among laboratory technicians compared to clerical personnel (42/50; 84% vs. 29/50; 58%; p = 0.002). Additionally, the mean Neck Disability Index (NDI) score was significantly higher in laboratory technicians (20.5 ± 8.4) than in clerical staff (16.4 ± 7.1; p = 0.01), indicating greater functional impairment in the former group.

**Table 2:** Prevalence of Neck Pain and Functional Disability Among Study Participants (N = 100) Data are presented as mean ± standard deviation or number (percentage). NDI: Neck Disability Index; χ²: chi-square test statistic; t: independent t-test statistic. A p-value of less than 0.05 was considered statistically significant.

Variable	Laboratory Technicians (n = 50)	Clerical Staff (n = 50)	Test Statistic	p-value
12-month neck pain prevalence, n (%)	42 (84%)	29 (58%)	χ² = 8.84	0.002
Mean NDI score ± SD	20.5 ± 8.4	16.4 ± 7.1	t = 2.64	0.01

The univariate analysis of occupational risk factors associated with neck pain is presented in Table 3. Individuals maintaining static postures for more than four hours daily demonstrated a significantly increased likelihood of neck pain (odds ratio (OR) = 3.2; 95% confidence interval (CI): 1.8-5.7; p < 0.001). Additionally, poor posture was significantly associated with a higher likelihood of neck pain (OR = 2.9; 95% CI: 1.6-5.2; p < 0.001). Moreover, participants who did not take regular work breaks had greater odds of neck pain compared to those who took breaks (OR = 3.3; 95% CI: 1.7-6.4; p = 0.002).

**Table 3:** Univariate Analysis of Occupational Risk Factors Associated With Neck Pain (N = 100) OR: odds ratio; CI: confidence interval. Univariate logistic regression analysis was performed. A p-value of less than 0.05 was considered statistically significant.

Risk Factor	OR	95% CI	p-value
Static posture >4 h/day	3.2	1.8-5.7	<0.001
Poor posture	2.9	1.6-5.2	<0.001
No regular breaks	3.3	1.7-6.4	0.002

The multivariate logistic regression analysis identifying independent predictors of neck pain is presented in Table 4. After adjusting for potential confounding factors, female sex (aOR = 2.4; 95% CI: 1.3-4.5; p = 0.007), maintaining a static posture for more than four hours daily (aOR = 4.1; 95% CI: 2.1-8.0; p < 0.001), a body mass index (BMI) exceeding 25 kg/m² (aOR = 1.9; 95% CI: 1.0-3.6; p = 0.04), and low ergonomic awareness (aOR = 2.2; 95% CI: 1.2-4.1; p = 0.01) were independently associated with neck pain.

**Table 4:** Multivariate Logistic Regression Analysis of Independent Predictors of Neck Pain (N = 100) aOR: adjusted odds ratio; CI: confidence interval. Multivariate logistic regression analysis was performed, including variables that were significant in the univariate analysis. A p-value of less than 0.05 was considered statistically significant.

Predictor	Adjusted OR (aOR)	95% CI	p-value
Female gender	2.4	1.3-4.5	0.007
Static posture >4 h/day	4.1	2.1-8.0	<0.001
BMI >25 kg/m²	1.9	1.0-3.6	0.04
Low ergonomic awareness	2.2	1.2-4.1	0.01

## Discussion

The overall 12-month prevalence of neck pain (71/100; 71%) observed in this study indicates a significant occupational health burden. Comparable high prevalence rates have been documented among healthcare and office personnel engaged in repetitive and static tasks [6]. The elevated prevalence identified herein underscores the growing impact of work-related neck pain within hospital environments, where prolonged, task-specific postures are often unavoidable.

Laboratory technicians demonstrated a significantly higher prevalence of neck pain compared to clerical staff, a disparity that may be related to sustained work-related postures such as prolonged cervical flexion and repetitive task performance. Biomechanical research indicates that prolonged cervical flexion increases compressive forces on cervical intervertebral discs and posterior anatomical structures, thereby predisposing individuals to chronic pain conditions [9,14]. Moreover, continuous visual concentration coupled with limited postural variability during laboratory procedures may intensify cumulative cervical strain over time.

The observed differences in age and work experience duration between laboratory technicians and clerical staff likely reflect the underlying institutional workforce structure rather than intentional selection bias. However, these baseline differences may have influenced the observed associations. Although multivariate analysis was conducted to adjust for potential confounders, residual confounding cannot be entirely ruled out.

Prolonged static posture exceeding four hours per day emerged as the strongest independent predictor of neck pain (adjusted odds ratio (aOR) = 4.1). This finding corroborates prior occupational studies demonstrating strong correlations between the duration of static neck postures and the onset of symptoms [8,15]. Sustained muscle activation without sufficient recovery intervals may induce ischemia, fatigue, and subsequent sensitization to pain.

Female gender was independently associated with an increased risk of neck pain (aOR = 2.4). Epidemiological evidence suggests that women may exhibit heightened susceptibility to work-related musculoskeletal conditions due to biomechanical and hormonal influences [10,16]. Variations in muscle endurance and pain perception thresholds may partially explain this association.

Elevated body mass index (BMI) was also identified as a significant predictor. Excess body weight can alter spinal biomechanics and contribute to muscular fatigue, thereby increasing vulnerability to neck pain [17]. Additionally, diminished physical activity levels commonly associated with higher BMI may further predispose individuals to discomfort.

Low ergonomic awareness was found to be significantly associated with neck pain. Structured ergonomic interventions, including workstation modifications and scheduled micro-breaks, have demonstrated efficacy in reducing such symptoms among office workers [10]. Consequently, the implementation of regular ergonomic training and institutional policies is recommended as a critical preventive measure.

The mean Neck Disability Index (NDI) score indicated moderate disability, reflecting a functional impact that extends beyond simple symptom reporting. Previous validation studies have demonstrated that even modest increases in NDI scores correspond with measurable functional limitations [18]. Although severe disability was uncommon among participants, persistent moderate impairment may still adversely affect productivity and work quality.

When compared to other hospital-based occupational groups, such as nurses and physiotherapists, the burden of neck pain among laboratory technicians appears comparable, underscoring the necessity for targeted preventive strategies [19]. Early identification of high-risk individuals and the establishment of structured occupational health surveillance programs may mitigate long-term complications. These findings highlight the need for ergonomic education, periodic posture correction initiatives, and administrative policies that promote regular rest breaks.

Limitations

The cross-sectional design of this study limits the ability to draw causal inferences; therefore, the identified associations should not be interpreted as causal relationships. Additionally, reliance on self-reported data may introduce recall and reporting biases, as well as potential misclassification of both exposure variables and outcome measures. The use of convenience sampling from a single center may introduce selection bias and limit the generalizability of the findings to other settings and populations. Differences in baseline characteristics between occupational groups may have influenced the observed associations despite adjustment through multivariate analysis, and residual confounding cannot be entirely excluded. Furthermore, certain exposure variables, such as posture and ergonomic awareness, were based on self-reported measures without detailed operational standardization, which may affect measurement validity. The distribution of participants across individual Neck Disability Index (NDI) categories was not analyzed, limiting a more detailed interpretation of functional disability levels. Future research should employ multicenter longitudinal designs, complemented by objective ergonomic assessments, to better establish causal relationships and long-term outcomes.

## Conclusions

Neck pain is commonly observed among laboratory technicians and clerical personnel and is significantly associated with factors such as sustained static postures and poor ergonomic conditions. These findings underscore the importance of workplace interventions, including ergonomic modifications, promotion of proper posture, and structured occupational health programs. Early identification of individuals at elevated risk, regular ergonomic training, and the incorporation of scheduled micro-breaks may contribute to improved occupational health and productivity in healthcare settings.
